# Persistent hypokalemia due to Conn’s syndrome resolved by robot-assisted laparoscopic adrenalectomy. A correct diagnostic approach for proper surgical therapy. Case report

**DOI:** 10.1093/jscr/rjaf446

**Published:** 2025-06-27

**Authors:** Santiago Muñoz-Palomeque, Miguel R Mesías, Diego M Mosquera, Fernando Jiménez Jaramillo

**Affiliations:** Department of General Surgery, Hospital Metropolitano, Quito, Ecuador, 170508; Faculty of Medical, Health and Life Sciences, Universidad Internacional del Ecuador, Quito, Ecuador, 170411; Department of Surgery, Division of General and Oncologic Surgery, Hospital Metropolitano, Quito, Ecuador, 170508; Department of Surgery, Division of General Surgery, Hospital Metropolitano, Quito, Ecuador, 170508; Department of Internal Medicine, Division of Nephrology, Hospital Metropolitano, Quito, Ecuador, 170508

**Keywords:** adrenalectomy, adrenocortical adenoma, adenoma, Conn, hypokalemia, robotic surgical procedures

## Abstract

Primary hyperaldosteronism, or Conn’s syndrome, is a leading cause of secondary hypertension, often presenting with persistent hypokalemia. We report the case of a 44-year-old female with refractory hypokalemia and hypertension, ultimately diagnosed with a unilateral aldosterone-producing adenoma. Diagnostic workup included elevated transtubular potassium gradient and aldosterone-to-renin ratio, confirmed by computed tomography imaging showing a typical left adrenal adenoma. The patient underwent robot-assisted laparoscopic adrenalectomy, resulting in normalized potassium levels and discontinuation of antihypertensive medications. Histopathology confirmed adrenal adenoma. This case underscores the importance of a structured diagnostic algorithm in detecting surgically correctable causes of secondary hypertension. Robotic-assisted surgery offered superior precision, minimal blood loss, and rapid recovery, proving especially advantageous in complex or reoperative cases. In resource-limited settings, where advanced technology is scarce, promoting diagnostic accuracy is vital for effective management. This case advocates for the broader adoption of minimally invasive approaches and encourages investment in surgical innovation in developing countries.

## Introduction

Currently, primary hyperaldosteronism is the most common hypertensive disorder of the adrenal cortex, with aldosterone-producing adenoma being the most common presentation [1–3].

Along these lines, unilateral aldosterone-producing adenoma, also known as Conn’s syndrome, represents a clinical syndrome characterized by hypertension and hypokalemia, with low or suppressed plasma renin activity and increased aldosterone excretion due to excess circulating aldosterone produced autonomously by a benign adrenal tumor, usually small, anterior, and solitary, located at the glandular margin. This tumor is more likely to be cured by unilateral adrenalectomy when aldosterone production is highly independent of renin-angiotensin and is lateralized to a specific adrenal gland [1–3].

We report the case of a female patient who presented with persistent hypokalemia and secondary arterial hypertension and was diagnosed with primary hyperaldosteronism due to a left adrenal adenoma (Conn’s syndrome). A robotic-assisted laparoscopic left adrenalectomy was performed with excellent results.

## Case report

**Figure 1:**
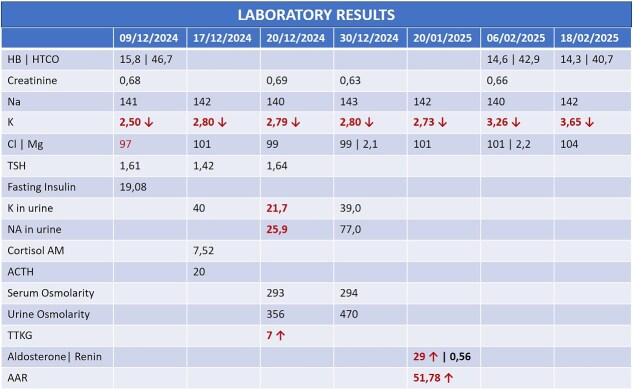
Laboratory history during the diagnostic process.

**Figure 2:**
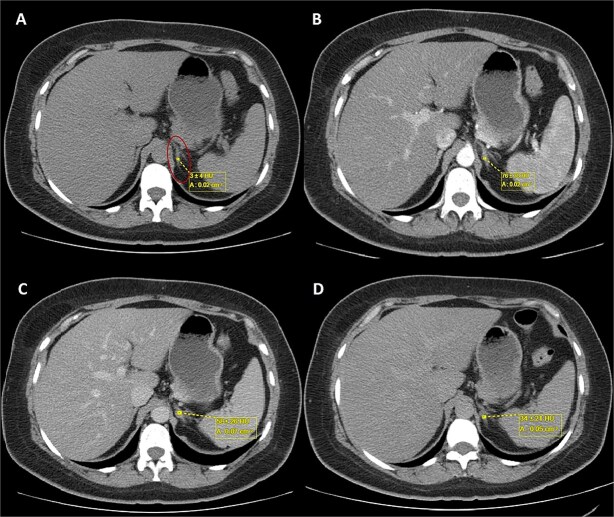
Triphasic abdominal CT scan with left adrenal adenoma (ellipse). (A) Simple phase, (B) arterial phase, (C) venous phase, (D) late venous phase.

**Figure 3:**
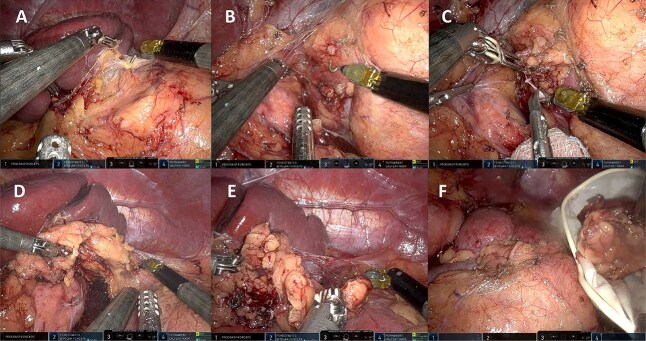
Left robotic adrenalectomy. (A, B) Left adrenal exposure, (C) control of the main adrenal vein, (D) gland dissection, (E) completion of the dissection, (F) end of the operation.

A 44-year-old female patient with a history of abdominoplasty and cesarean section presented with a long-standing history of unexplained headache and nocturnal sweating, accompanied by fatigue and adynamia. The evaluation revealed elevated blood pressure and persistent moderate hypokalemia that did not respond to clinical management. A diagnostic approach was completed to determine the trans tubular potassium gradient (TTKG) and renin/aldosterone ratio (AAR), with elevated values ​​suggesting primary hyperaldosteronism (Fig. 1). An abdominal computed tomography (CT) scan revealed findings compatible with left adrenal adenoma (Fig. 2). A robotic-assisted laparoscopic left adrenalectomy was performed (Fig. 3), finding a left adrenal gland ⁓4 × 4 cm in diameter with a tumor ⁓2 cm in diameter attached to it. The procedure, which lasted 120 min and produced a minimal amount of blood loss of 10 mm, was uneventful. Following successful postoperative progress, the patient was discharged on the second postoperative day.

The histopathology results confirmed the diagnosis of adrenal adenoma, consisting of a proliferation of uniform nests of cells with ample, clear, foamy cytoplasm and central nuclei without atypia, pleomorphism, or mitosis. Delimited by a thin fibrous capsule, the gland was non-tumorous and showed no significant alterations.

During follow-up in the outpatient clinic, the patient’s potassium and blood pressure were trending toward normal levels, and she was no longer on antihypertensive medications.

## Discussion

Patients with an adrenal incidentaloma 1 cm or larger in diameter should undergo biochemical testing and imaging, with adrenal CT being appropriate for stratifying the risk of malignancy and suspected pheochromocytoma [4].

**Figure 4:**
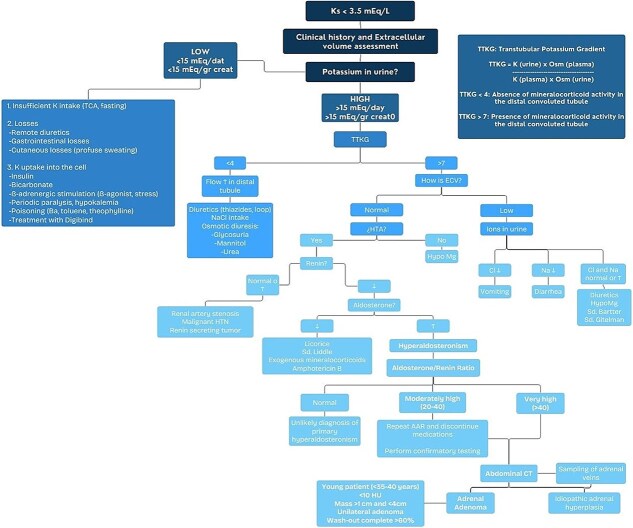
Diagnostic flowchart of primary hyperaldosteronism from hypokalemia. Based on the algorithms proposed by de Sequera Ortíz *et al.* [5] and Ares *et al.* [6].

In patients presenting with persistent hypertension or hypokalemia, primary hyperaldosteronism should be considered. The initial diagnostic workup should include TTKG and ARR. An elevated ARR, especially in the setting of suppressed renin and normal extracellular volume, strongly suggests autonomous aldosterone production. Bilateral adrenal vein sampling and imaging, particularly contrast-enhanced CT, can help localize a functioning adenoma and differentiate it from idiopathic adrenal hyperplasia (Fig. 4) [4–8].

In this case, elevated TTKG and ARR, along with imaging findings [size <4 cm with tumor with regular borders without calcifications, low attenuation <10 Hounsfield Units (HU), high uptake in the arterial phase, lower uptake in the venous phase, and absolute wash-out >60%] (Fig. 2), supported the diagnosis of a left-sided aldosterone-producing adenoma. Surgical resection remains the treatment of choice for unilateral disease, offering a potential cure [9, 10].

Minimally invasive adrenalectomy is now the gold standard for benign adrenal tumors under 6 cm treatment, while open surgery is generally reserved for tumors larger than 7–8 cm [4, 11–14]. Robotic-assisted approaches offer distinct advantages over traditional laparoscopy, including improved dexterity, visualization, and reduced blood loss—benefits especially relevant in technically demanding cases or reoperations [14–17]. In our case, robotic surgery allowed for precise dissection, minimal bleeding, and rapid recovery.

Minimally invasive adrenalectomy can be performed via an anterior, thoracoabdominal, or retroperitoneal approach, depending on the surgeon’s experience, tumor location and size, and patient characteristics [4, 13].

Regardless of the side of the pathological gland, there are five steps to complete adrenalectomy: right/left exposure, control of the main adrenal vein, gland dissection, completion of the dissection, and end of the operation [17], which is summarized in the figures of our surgical procedure (Fig. 3).

## Conclusions

Primary hyperaldosteronism due to a unilateral adrenal adenoma (Conn’s syndrome) should be a key consideration in patients with unexplained hypokalemia and hypertension. A structured diagnostic approach ensures accurate identification of surgically correctable causes, for which the diagnostic algorithm presented in this article is very convenient.

Robotic-assisted adrenalectomy is a safe, minimally invasive option with excellent outcomes, particularly in appropriately selected patients. This case reinforces the value of early diagnosis and modern surgical techniques, even in resource-limited settings. Promoting access to robotic technology and training may improve outcomes in endocrine surgery across the globe.
